# Dietary composition influences sperm quality and testis damage via endoplasmic reticulum stress in lambs

**DOI:** 10.1002/vms3.1504

**Published:** 2024-06-16

**Authors:** Aybuke Imik, Murat Eren, Mazhar Burak Can, Seckin Ozkanlar, Ali Dogan Omur, Mehmet Akif Aydin, Serhat Sunar, Serkan Ali Akarsu

**Affiliations:** ^1^ Department of Nutrition and Dietetics Institute of Health Sciences Selçuk University Konya Turkey; ^2^ Department of Animal Nutrition and Nutritional Diseases Institute of Health Sciences Erciyes University Kayseri Turkey; ^3^ Department of Animal Nutrition and Nutritional Diseases Institute of Health Sciences Atatürk University Erzurum Turkey; ^4^ Department of Biochemistry Institute of Health Sciences Atatürk University Erzurum Turkey; ^5^ Department of Reproduction and Artificial Insemination Institute of Health Sciences Atatürk University Erzurum Turkey; ^6^ Department of Reproduction and Artificial Insemination Institute of Health Sciences Kafkas University Kars Turkey

**Keywords:** antioxidant, gluten, protein expression, sperm quality

## Abstract

**Background:**

The metabolic impacts of including soya meal, wheat gluten and corn gluten in the diet of male lambs could influence their reproductive performance.

**Objectives:**

An experiment was carried out to assess the effects of corn gluten, wheat gluten and soya meal on the reproductive system of male lambs.

**Methods:**

Twenty‐four male Morkaraman lambs, aged 9 months, were utilized in this study and were fed experimental diets for 56 days. The lambs were divided into a control group (soybean meal + safflower meal), a corn group (corn gluten) and a wheat group (wheat gluten).

**Results:**

The serum follicle‐stimulating hormone level of the control group was significantly higher and tumour necrosis factor‐alpha (TNF‐α) level was lower than the wheat and corn gluten groups (*p *< 0.05). The lowest malondialdehyde level in testicular tissue was observed in the control group, whereas the highest was in the wheat gluten group (*p *< 0.05). The glutathione level in the control group was significantly higher than in the other groups (*p *< 0.05). The corn gluten group showed the highest CHOP and IRE1 levels; the lowest Bcl‐2 levels and the highest IL‐1B and P2 × 7R levels were found in the wheat group; and the lowest TNF‐α levels were in the control group (*p *< 0.05). Additionally, the study revealed that diet had a significant impact on spermatological parameters of the testis such as diameter, volume and weight (*p *< 0.05).

**Conclusions:**

These results concluded that the inclusion of different protein sources in the diet of reproductive male lambs affects the metabolism of testicular tissue.

## Graphical abstract

In this study, the effects of different protein sources on the reproductive system of male lambs were investigated. It was observed that the protein sources used in the diet affected the serum FSH and TNF‐α levels, as well as the MDA and GSH levels of testis tissue. Additionally, the protein expression markers CHOP, ATF6, TNF‐α, Bcl‐2, IL‐1B and P2 × 7R were impacted in varying ways. These findings suggest that misfolded proteins may lead to ER stress through different mechanisms.

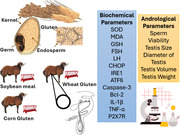

## 1 INTRODUCTION

The nutritional value of feed is influenced not just by the nutrient ratio but also by how these nutrients are metabolized within the organism's cells. Soybean meal and corn gluten are frequently utilized as protein sources in animal nutrition, whereas wheat gluten is not. Additionally, it is recognized that the chemical composition and metabolism of these protein sources vary.

It is well established that nutrition has a significant impact on hormonal metabolism (Gumus et al., 2020; Imik, 2019; Imik et al., 2023; Qi et al., 2019; Ye et al., 2022). Follicle‐stimulating hormone (FSH) is released from the pituitary gland in the brain and travels to the testicle through the bloodstream. FSH activates the Sertoli cells, Leydig cells and sperm stem cells in the testis for the maturation of sperm cells. Leydig cells are responsible for testosterone production, and if they do not function properly, testosterone levels decrease. It is well known that FSH directly affects the sperm stem cells and contributes to their maturation (Ding et al., 2022; Eisenberg et al., 2023). Luteinizing hormone (LH) is a glycoprotein produced in the anterior lobe of the pituitary gland. LH stimulates the production of the hormone testosterone in males and regulates reproductive and sexual functions (Ding et al., 2022; Eisenberg et al., 2023; Kivrak & Aydin, 2023). Overfeeding, underfeeding or malnutrition of breeding animals negatively affects the reproductive system. Therefore, balanced nutrition for breeding animals is crucial for the regular and long‐term release of sex hormones (Skoracka et al., 2020; Yildiriret al., 2022).

In living organisms, free radicals formed at the end of metabolic activities are removed from the organism by the antioxidant system. Antioxidant metabolism develops an intensive defense system in many tissues and organs in the organism, especially in the liver (Elmas et al., 2022; Gumus et al., 2017; Haliciet al., 2012; Valko et al., 2007). The most important parameters used to determine antioxidant metabolism in the body are malondialdehyde (MDA), superoxide dismutase (SOD) and glutathione (GSH). Antioxidant defense system becomes inadequate as a result of excessive increase in free radicals formed in the body and causes lipid peroxidation (Halliwell, 1999; Halliwell & Chirico, 1993). The enzyme SOD is an important antioxidant defense system that converts free or superoxide radicals that cause cell damage into less reactive hydrogen peroxide and oxygen. Because of this reason, increased free radicals as a result of decreased SOD activity lead to cell damage (Halliwell, 1999; Halliwell & Chirico, 1993). GSH is responsible for the protection of antioxidant molecules in the cell, prevention of oxidative damage and inactivation of drugs (Meister, 1991).

Mammalian metabolic activities in tissue cells vary depending on many factors. Nutrition is one of these factors. CHOP, IRE1, ATF6, Caspase‐3, Bcl‐2, IL‐1B, P2 × 7R and tumour necrosis factor‐alpha (TNF‐α) are the most important parameters that are used to detect protein expression in tissue cells. In tissue cells, the activation of parameters such as CHOP, IRE1 and ATF6 induces endoplasmic reticulum (ER) stress (Stauffer, 2020). Bcl‐2 is a protein known for inhibiting apoptosis in cells. Caspase‐3 increases cell susceptibility to apoptosis (Kaya, 2012). IL‐1B, P2 × 7R and TNF‐α serve as markers of inflammation in cells (Gumus et al., 2021). Gao et al. (2022) found that herbicides induce cellular DNA damage in grass carp liver through the NO/iNOS/NF‐κB pathway, which can be partially mitigated by tannic acid. Wang et al. (2024) reported that the imidacloprid insecticide triggers mitochondrial apoptosis (Caspase‐3, Caspase‐9, Bax and Cyt‐c), necroptosis (Caspase‐8, RIPK1, RIPK3 and MLKL) and immune dysfunction in chickens by activating the key components of the mitogen‐activated protein kinase signalling pathway, resulting in oxidative stress. They also observed that these dysfunctions are significantly reduced by the polyphenolic compound Resveratrol. Lv et al. (2023) demonstrated that selenium alleviates pyroptosis and inflammation in grass carp CIK cells through the IRAK1/TAK1/IKK pathway induced by lead. Lei et al. (2024) showed that selenomethionine modulates the JAK2/STAT3/A20 pathway via oxidative stress to alleviate LPS‐induced pyroptosis and inflammation in chicken hearts. In the present study, the above‐mentioned parameters were analysed in testicular tissue to determine the effect of different protein sources added to the ration on protein expression.

It was aimed to investigate the effect mechanism of different protein sources added to male lamb diets on serum hormonal metabolism and testicular tissue via antioxidant and ER stress.

## 2 MATERIALS AND METHODS

### 2.1 Animals and experiment design

In this study, 24 Morkaraman males aged 9 months were used. The subjects were divided into three groups: control, wheat and corn groups, with corresponding feeds detailed in Table 1. All groups received diets with identical protein content (17% HP) and energy level (ME: 2700 kcal/kg) for 56 days. After the experiment, serum samples were collected from the animals’ blood tissue, and the testicular tissue of the slaughtered animals was preserved at −80°C for analysis.

**TABLE 1:** Composition of lamb fattening feed used in the study.

Item	Control	Wheat G	Corn G
Ingredients, %			
Barley	60.00	52.50	60.00
Soybean meal	15.93		
Rice bran	10.00		
Safflower meal	7.47		
Wheat		30.00	
Corn			18.22
Corn gluten			14.78
Wheat gluten		10.30	
Molasses	3.00	3.00	3.00
Marble dust	2.40	1.65	2.35
Soy oil	0.60	0.33	
Salt	0.30	0.30	0.31
Ammonium chloride	0.20	0.30	0.28
Dicalcium phosphate		1.51	0.96
Vitamin–mineral premix	0.10	0.10	0.10
Total	100	100	100
Nutrient composition			
Crude protein, %	17.00	17.00	17.00
Metabolizable energy (kcal/kg)	2.700	2.700	2.700

### 2.2 Biochemical analysis

#### 2.2.1 Preparation of tissues

PBS‐washed testicular tissue samples were lysed with Qiagen Tissue Lyser II at 30 hz for 3 min by adding liquid nitrogen. Afterwards, 0.1 g of tissue samples were homogenized for 30 s at 30 hz in Tissue Lyser II by adding homogenate buffers.

#### 2.2.2 Assessment of oxidant/antioxidant parameters

Following the preparation of testicular tissues, the absorbances of all oxidants and antioxidants in each sample were determined using a spectrophotometer (ELISA reader, BioTek, μQuant) with specific protocols. The chemicals from Sigma‐Aldrich Company were utilized for the analyses.

#### 2.2.3 Superoxide dismutase (SOD) enzyme activity measurement

This method is based on the enzymatic process; it reduces free radicals in the presence of nitroblue tetrazolium in the sample, and the enzyme SOD inhibits the free radicals. A spectrophotometer set at 560 nm measures the colour change caused by the reaction (Sun et al., 1988).

#### 2.2.4 Measurement of MDA levels

MDA is determined by measuring the absorbance of the pink molecule that results from the interaction of MDA with thiobarbituric acid at a wavelength of 532 nm (Ohkawa et al., 1979).

#### 2.2.5 Measurements of tissue GSH levels

The measurement of GSH is based on Sedlak and Lindsay (1968). Accordingly, it is based on the measurement of the absorbance at 412 nm of the bright yellow compound given by GSH with 5,5′‐dithiobis‐(2‐nitrobenzoic acid) (Sedlak & Lindsay, 1968).

#### 2.2.6 Serum FSH, LH and TNF‐α analysis

Serum ELISA analyses were done with FSH (BT Lab Sheep Cat: No: E0166Sh), LH (BT Lab Sheep Cat: No: E0106Sh) and TNF‐α (AFG Sheep Bioscience Cat: No: EK770774) kits by ELISA method in Eliza Reader device (BioNTech) according to the manufacturer's protocol.

#### 2.2.7 Western blot analysis

The testis tissue samples were used to determine relative protein expressions of Chop, IRE1, ATF6, Caspase‐3, Bcl‐2, IL‐1B, TNF‐α and P2 × 7R. The tissue samples were crushed in nitrogen gas, and total protein was extracted from the tissue samples. The proteins were separated by 10% SDS–PAGE and transferred to the PVDF membrane. The membranes were incubated with 5% bovine serum albumin for blocking and then incubated with primary antibodies ((CHOP, Affinity, AF5280), (IRE1, Affinity, DF7709, dilution 1:1000), (ATF6, Affinity, DF6009, dilution 1:1000), (P2 × 7R, Proteintech, 11144‐1‐AP, dilution 1:1000), (Caspase‐3, Santa Cruz, sc‐56053, dilution 1:1000), (Bcl‐2, Santa Cruz, sc‐7382, dilution 1:1000), (IL‐1β, Affinity, AF5103, dilution 1:1000), (TNF‐α, Affinity, AF7014, dilution 1:1000) and beta‐actin (Santa Cruz, sc‐47778, dilution 1:1000)) at 4°C overnight. After washing the PVDF membranes, they were incubated with the HRP‐conjugated second antibody (Santa Cruz, sc‐2004/sc‐2005) for 90 min at room temperature. Then, the protein bands were visualized with the chemiluminescence reagent (Thermo, 3405) using Image Lab software from Bio‐Rad Laboratories (Bio‐Rad) and analysed by the ImageJ analyse program.

### 2.3 Andrological analysis

Among the testicular measurements, testicular diameter was measured with the help of a tape measure, whereas testicle diameter was measured with the help of a digital caliper (Gundogan et al., 2022). To measure the volume of double testicles including the scrotum, a 2‐L container was filled with water at 37°C, and the scrotum was immersed in this container. The water overflowing from the container was taken into a larger container kept at the bottom. The water taken was measured in a measuring cylinder and recorded as scrotum volume (Gundogan et al., 2003). Total testicular weight was calculated by weighing the testicles separated from the epididymis.

Eosin–nigrosin staining method was used to determine sperm viability rate. Briefly, a drop of eosin nigrosin dye was poured onto the sperm sample and mixed. The viability of the cells was evaluated by counting 200 cells under a phase contrast microscope. Sperm cells that absorbed eosin were considered dead (Ozturk et al., 2020).

### 2.4 Statistical analysis

Statistical analysis was performed using the SPSS package program (IBM Inc). The Kolmogorov–Smirnov test was used to determine the data's normality. A one‐way ANOVA test and post hoc Duncan test were performed to compare groups. *p* Values less than 0.05 at the 95% confidence interval were considered statistically significant. The data are presented as mean ± standard deviation from eight independent experiments (*n* = 8). Significance was denoted by different letters of the alphabet (a–c) for values with *p *< 0.05. Conversely, identical alphabet letters indicate nonsignificant differences between the groups (*p *> 0.05).

## 3 RESULTS

### 3.1 Tissue oxidative stress parameters

**FIGURE 1:**
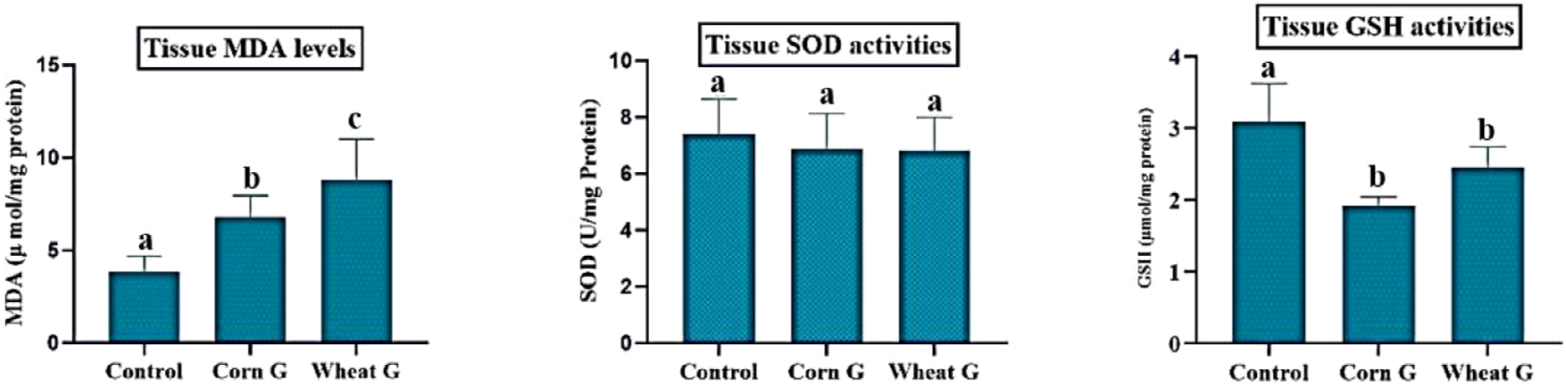
Biochemical tissue malondialdehyde (MDA) levels and superoxide dismutase (SOD) and glutathione (GSH) activities. The letters (a and b) indicate the statistical differences between the groups.

In analysis of tissue antioxidant and oxidant parameters, MDA levels were significantly increased in Corn G and Wheat G groups compared to the control group (*p *< 0.05). On the other hand, there was no significant difference between the groups in tissue SOD activity (*p *> 0.05). Tissue GSH activities showed that Corn G and Wheat G levels were significantly decreased compared to the control group (*p *< 0.05). The tissue MDA levels and tissue SOD and GSH activities of all groups are presented in Figure 1.

### 3.2 Serum FSH, LH and TNF‐α parameters

**FIGURE 2:**
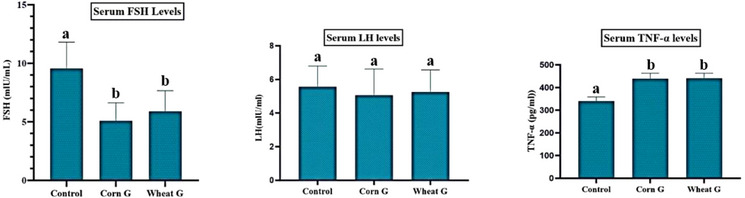
The serum concentration of follicular stimulant hormone (FSH), luteal hormone (LH) and tumour necrosis factor‐alpha (TNF‐α). The letters (a and b) indicate the statistical differences between the groups.

It was determined that serum FSH level was highest in the control group and this level decreased significantly in the Corn G and Wheat G groups (*p *< 0.05). No significant difference was found in serum LH level between the groups (*p *< 0.05). TNF‐α level, which is a serum inflammation parameter, was significantly increased in Corn G and Wheat G groups compared to the control group (*p *< 0.05). Serum FSH, LH and TNF‐α levels of all groups and their comparisons are presented in Figure 2.

### 3.3 Western blot analysis results

**FIGURE 3:**
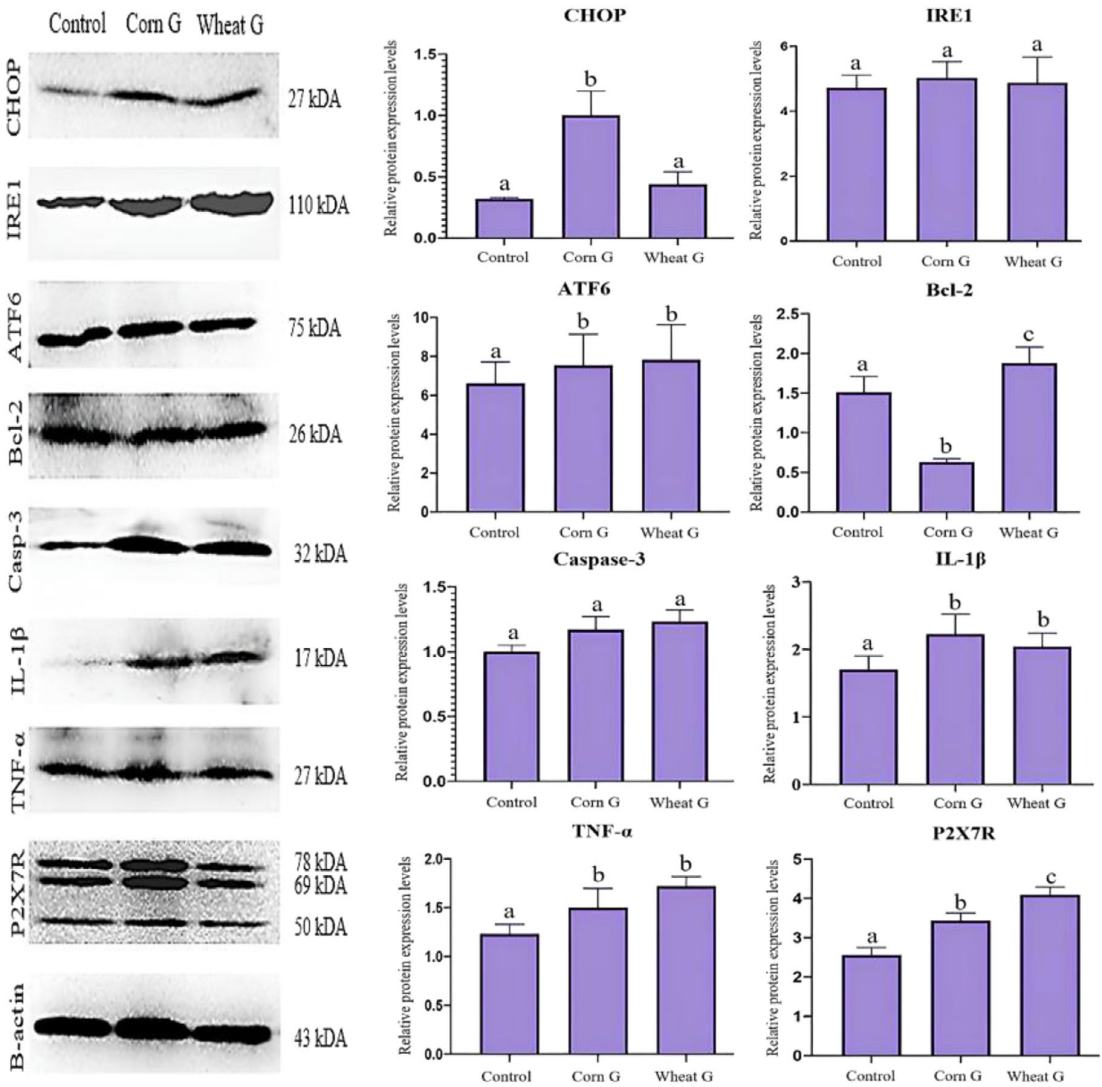
The relative expression levels of CHOP, IRE1, ATF6, Bcl‐2, Caspase‐3, IL‐1B, tumour necrosis factor‐alpha (TNF‐α) and P2 × 7R proteins, all expression normalized according to beta‐actin.

According to the results of our tissue protein analysis, there was a significant increase in CHOP and IRE1 protein expression levels in the Corn G group (*p *< 0.05). Wheat G group was similar to control (*p *> 0.05). Regarding ATF6 protein expression, there was an increase in Corn G and Wheat G groups, but the difference with the Control group was not significant (*p *> 0.05). Bcl‐2 expression was found to be significantly decreased in the Corn G group compared to the control and Wheat G groups (*p *< 0.05). Moreover, there was a significant increase in Caspase‐3 expression in the Corn G group compared to the other groups (*p *< 0.05). Regarding inflammation parameters, the highest IL1B, TNF‐α and P2 × 7R protein expressions were found in the Wheat G group. In Corn G group, the increase in TNF‐α and P2 × 7R proteins was found to be significant compared to the control group (*p *< 0.05), but there was no significant difference between the expressions of IL‐1B protein in Control and Corn G groups (*p *> 0.05). The expressions and expression levels of all proteins are shown in Figure 3.

### 3.4 Andrological findings

No statistically significant difference was found between the experimental groups in terms of sperm viability and testicular length. Testis diameter length was found to be higher in wheat gluten and corn gluten groups than in the control group (*p *< 0.05). Although testicular volume had the highest value in the wheat gluten group, a statistically significant difference was observed between it and the other experimental groups (*p *< 0.05). Testis weight was found to be higher in the corn gluten and wheat gluten groups compared to the control group (*p *< 0.05). The effect levels of different proteins on andrological parameters of the testis are shown in Table 2.

**TABLE 2:** Effect of different proteins used in the experiment on anthological parameters of the testis.

Groups	Sperm viability	Testis size	Diameter of testis	Testis volume	Testis weight
Control	40.83 ± 12.81	12.83 ± 1.52	24.16 ± 2.48^a^	196.66 ± 40.82^a^	165.83 ± 12^a^
Wheat G	38.75 ± 8.76	13.75 ± 1.38	27.75 ± 2.54^b^	305.62 ± 89.54^b^	215 ± 25.49^b^
Corn G	38.33 ± 7.52	13.00 ± 0.89	26.83 ± 1.83^ab^	235.83 ± 21.07^ab^	233.33 ± 25.23^b^

*Note*: The letters (a and b) were considered significant in terms of statistical difference (*p* < 0.05).

## 4 DISCUSSION

It is crucial to elucidate the metabolic activities in the cell to develop effective nutritional strategies for animals. This study was conducted to determine the metabolic effects of different protein sources by determining FSH and LH hormones and TNF‐α levels in serum tissue and antioxidant and protein expression markers in testicular tissue.

It is well known that balanced nutrition is very important in the development of the reproductive system (Gumus et al., 2020; Imik, 2019; Imik et al., 2023; Qi et al., 2019; Ye et al., 2022). Ye et al. (2022) reported that folic acid added to the feed of aged roosters (broilers) improved semen quality and spermatogenesis. Qi et al. (2019) reported that flax seed added to the feed of aged rooster (broiler) significantly increased FSH and LH levels. Additionally, they reported that it significantly affected spermatogenesis. Gumus et al. (2020) reported that wheat gluten, corn gluten and soybean meal added to the feed of male rats influenced sperm quality. Imik (2019) reported that wheat gluten fed to female rats from 20 days of age to 65 days of age significantly increased FSH and LH levels in serum and ovarian tissue when compared to the groups fed corn gluten and soybean meal. On the other hand, Imik et al. (2023) reported that wheat gluten fed from 21 days of age to 185 days of age resulted in an increase in FSH levels of serum tissue and LH levels of ovarian tissue in the groups fed corn gluten and soybean meal, whereas FSH and LH levels of ovarian tissue were not influenced. In our study, serum FSH levels were significantly higher in Control G than in Wheat G and Corn G, whereas LH levels were similar between the groups. This shows that different protein sources in the ration affect FSH release but have no effect on LH release. As a matter of fact, it is understood that the findings obtained in this study are similar to the information in the literature (Gumus et al., 2020; Imik, 2019; Imik et al., 2023; Qi et al., 2019; Ye et al., 2022).

It is known that antioxidant metabolism is shaped in other tissues and organs in the body, especially in liver tissue (Gumus et al., 2017; Halici et al., 2012). Tang et al. (2019) reported that polyunsaturated fatty acids included in the feed had an effect on the antioxidant metabolism of testicular tissue. Bas et al. (2023) reported that vitamin E, Zn and Se minerals added to the feed of Turkish geese at different ratios positively improved SOD, catalase, GSH peroxidase and GSH‐*S*‐transferase activities in testicular tissues but had no effect on MDA levels. Jiang et al. (2019) reported that curcumin which contains phenolic substance added to the feed of sheep had no effect on SOD in antioxidant parameters in serum tissue, whereas it had a significant effect on GPX. Bakir et al. (2020) reported that cherry extract (Cherry laurel = Laurocerasus officinalis Roem.) was given to rats with damaged testicular tissue for 8 weeks‐maintained oxidant‐antioxidant balance. Jiang et al. (2021) reported that corn gluten meal fed to weaned calves with different processes and additives affected their antioxidant metabolism. In our study, there was a significant difference between the MDA levels of the testicular tissue of the research groups. MDA level was highest in Wheat G group and lowest in Corn G group which was given soybean meal. It was determined that the effect of protein sources on oxidative stress in testicular tissue was different, and this effect was highest in the group consuming wheat gluten (*p *< 0.05). On the other hand, GSH level was higher in the control group compared to the groups consuming corn and wheat gluten, indicating that feed content was effective on this parameter (*p *< 0.05). As SOD activity in testicular tissue was similar between the groups, it can be stated that it was not affected by different protein sources. The findings of this study demonstrate that corn gluten, wheat gluten and soybean meal affect the antioxidant metabolism of testicular tissue. Differences in the chemical composition of the protein sources included in the ration cause variable metabolic effects. These results show that it may vary depending on the nutritional factor as stated in the literature studies (Bakir et al., 2020; Bas et al., 2023; Gumus et al., 2017; Halici et al., 2012; Jiang et al., 2021, 2019; Tang et al., 2019). This suggests that dietary strategy is important for healthy eating.

Many metabolic activities take place in the cells of living organisms that are known or not fully elucidated. This metabolic activity in tissue cells varies depending on many factors. One of these factors is nutrition. Tang et al. (2019) reported that polyunsaturated fatty acids included in the feed of animals affect cellular functions and gene expression by playing an important role in the composition of membranes in cells, protecting haemostasis, affecting membrane fluidity, affecting cellular signalling processes and affecting cellular functions and gene expression. Jiang et al. (2019) reported that the effect of curcumin which contains phenolic substance and added to the feed of sheep on Bcl‐2 in the testicular tissue was dose associated and the effect of 450 mg was limited to the control group, whereas the effect of 900 mg was significant. In contrast to Bcl‐2, they reported that the effect on Caspase‐3 decreased depending on the dose. Ozkaraca et al. (2021) reported that Caspase‐3‐associated apoptosis was observed in nerve cells of cows, horses, donkeys, dogs and cats naturally infected with rabies. In addition, they also reported that Caspase‐3 down‐regulated the expression of Caspase‐3 and up‐regulated the expression of bcl‐2. In a study on patients (with neuroinflammation) and healthy people, Bobińska et al. (2016) found that TNF gene expression increased significantly with degenerative disorders. At the same time, they also reported that patients’ memory and memory impairment, attention disorders and decreased learning capacity. Ye et al. (2022) reported that folic acid significantly affected protein expression in the study they conducted by adding folic acid to the feed of old roosters (broilers). Qi et al. (2019) reported that flax seed added to the feed of aged rooster (broiler) affected gene expression. Gumus et al. (2021) reported IL‐1β and TNF‐α levels of soybean meal, corn and wheat gluten fed to rats as 55.83, 81.65 and 72.78, 46.37, 61.95 and 61.31, respectively. Bakir et al. (2020) reported that cherry extract (Cherry laurel = Laurocerasus officinalis Roem) given to rats with damaged testicular tissue for 8 weeks provided sperm DNA integrity. In this study, the corn gluten group significantly decreased the Bcl‐2 level when compared to the soybean meal and wheat gluten groups (*p *< 0.05), indicating that the cells were more prone to apoptosis. IL‐1B and P2 × 7R levels of wheat gluten group were higher than control and corn gluten groups, indicating that ration wheat gluten stimulates cells more and may cause inflammation. TNF‐α levels of corn and wheat gluten groups were higher than the control group, indicating that corn and wheat gluten stimulate testicular tissue more against this parameter. Moreover, TNF‐α levels of serum and testicular tissues were similar, indicating that corn and wheat gluten stimulated the tissues more. The results of this study suggest that the protein sources used as the main nutrients in the diet affect protein expression markers at different rates. These findings indicate that feeding affects protein expression and this information agrees with the reports in the literature.

ER stress is a condition where the ER, which plays a critical role in protein synthesis, folding and processing, becomes overloaded or dysfunctional, accumulating misfolded or unfolded proteins (Gelen et al., 2024). High dietary protein intake might overload the ER's capacity to fold properly and process proteins, accumulating misfolded proteins and triggering the unfolded protein response (UPR) (Li et al., 2014). Additionally, the composition of nutritional proteins is critical, and an imbalance in essential amino acids might lead to incomplete or faulty protein synthesis, resulting in ER stress (Lemmer et al., 2021). Latest studies emphasize that ER stress has emerged as an important molecular mechanism in tissue injury (Han et al., 2021). ATF6 is one of the main transmembrane proteins that initiate UPR signalling in the ER stress response. ATF6 is activated by dissociating from GRP78 when ER stress is triggered. After dissociating with GRP78, ATF6 is transported to the Golgi in response to ER stress. Activated ATF6 then migrates to the nucleus where it induces many proapoptotic genes, including CHOP (Han et al., 2021). CHOP activates the mitochondrial apoptosis pathway (Chong et al., 2017). A study showed that low‐dose radiation induced ROS‐mediated ER stress and apoptosis in testicular cells of mice and that the ER stress mechanism was regulated by IRE1, PERK and ATF6 pathways (Wang et al., 2013). In another study, it was found that apoptosis was induced by activation of ER stress markers CHOP in mouse Leydig cells (Lin et al., 2015). Although ATF6 was similar in all groups in the study, which indicated that there was no effect of ration, CHOP and IRE1 levels of the group given corn gluten were significantly higher than the group given wheat gluten and soybean meal (*p *< 0.05). It is seen that the testicular cells (ER) of animals eating corn gluten are stressed.

For rams to be used as breeding stock, testicular morphometric measurements are required to be at optimum values. There is a positive correlation between fertility and testicular morphometry in rams (Aksoy et al., 1994). Various results have been obtained in unilateral length measurement of the testicle and testis volume (Demirci, 1993; Kaya et al., 1999). However, in the double‐sided measurement we made in our study, testicular length and testicular volume were observed to increase in the other experimental groups compared to the control group (*p *< 0.05). Various results have been obtained in unilateral length measurement of the testicle. However, in the double‐sided measurement we made in our study, testicular length was observed to increase in the other experimental groups compared to the control group (*p *< 0.05). It is thought that this situation arises from the relationship between testicular sizes and nutrition.

Sperm viability is one of the important fertility indicators (Ceribasi et al., 2020). Studies show that there may be a relationship between nutrition and sperm viability (Matos et al., 2023; Zhao et al., 2017). In the presented study, no statistical difference was observed between the groups in terms of sperm viability. It is considered that this data may be related to the optimal nutrition of the animals in the experimental groups.

## 5 CONCLUSIONS

Although wheat gluten included in the diets of male lambs influenced serum FSH and TNF‐α levels, soybean meal + safflower meal, corn gluten and wheat gluten influenced testicular tissue MDA levels to different degrees, and GSH levels were highest in the control group, which indicates that diet affects reproductive performance. Furthermore, testicular tissue protein expression markers CHOP, IRE1, Bcl‐2, IL‐1B and P2 × 7R and TNF‐α levels were influenced differently by the protein sources used in the diet, showing that unfolded or misfolded protein accumulations that cause ER stress use different pathways. These findings are expected to play a crucial role in determining optimal feeding strategies for animals. Further research is necessary to explore the impact of nutrition on protein expression in tissues.

## AUTHOR CONTRIBUTIONS

All authors contributed to the conception and design of the study. Material preparation and data collection were carried out by Mazhar Burak Can, Aybuke Imik and Murat Eren. Biochemical and andrological analyses were performed by Seckin Ozkanlar, Ali Dogan Omur, Mehmet Akif Aydin, Serhat Sunar and Serkan Ali Akarsu. The first draft of the manuscript was written by Mazhar Burak Can, Aybuke Imik, Murat Eren, Seckin Ozkanlar and Ali Dogan Omur and all authors commented on previous versions of the manuscript. All authors read and approved the final manuscript.

## CONFLICT OF INTEREST STATEMENT

The authors declare that they have no conflicts of interest.

## FUNDING INFORMATION

No funding was received to assist with the preparation of this manuscript.

### ETHICS STATEMENT

Ethics committee approval for this study was obtained from Atatürk University, Faculty of Veterinary Medicine, Unit Ethics Committee on 22/02/2022 with decision number 2022/6.

### PEER REVIEW

The peer review history for this article is available at https://www.webofscience.com/api/gateway/wos/peer‐review/10.1002/vms3.1504.

## Data Availability

The data used to support the findings of this study are included within the article.
